# Repetitive sampling inspection plan for cancer patients using exponentiated half-logistic distribution under indeterminacy

**DOI:** 10.1038/s41598-023-40445-6

**Published:** 2023-08-23

**Authors:** Gadde Srinivasa Rao, Peter Josephat Kirigiti

**Affiliations:** Department of Mathematics and Statistics, The University of Dodoma, P.O. Box: 259, Dodoma, Tanzania

**Keywords:** Statistics, Cancer, Oncology, Mathematics and computing

## Abstract

This piece of work deals with a time truncated sampling scheme for cancer patients using exponentiated half-logistic distribution (EHLD) based on indeterminacy. We have studied time truncated schemes like repetitive acceptance sampling plan (RASP) under indeterminacy. We have estimated the projected scheme parameters such as sample size and acceptance and rejection sample numbers for known indeterminacy parameters. In addition to the projected sampling scheme quantities, the corresponding tables are generated for various values of indeterminacy parameters. The results of a sampling scheme show that the average sample number (ASN) decreases as indeterminacy values increase. It leads that the indeterminacy parameter is played a crucial portrayal in ASN. A comparative study is carried out with existing sampling schemes based on indeterminacy and classical sampling schemes. The evaluated sampling schemes are exemplified with the help of cancer data. From tables and exemplification, we wind up that the projected RSP scheme under indeterminacy desired a smaller sample size than the existing schemes.

## Introduction

Cancer, one of the most severe and lethal diseases, necessitates aberrant cell development that intensifies. It is a cancerous tumor with irregular cell proliferation that has the potential to attack or spread to other human body organs. For further information, check^1^. The rise may also be immediate, passing directly through the blood or lymphatic system. Different organs may be affected by cancer, and each type of cancer has unique traits. In relation to the location of cancers, various types of cancer are identified, including cervical cancer, lung cancer, gynecological cancer, skin cancer, brain cancer, breast cancer, etc. Breast cancer is the most common type of cancer. In recent years, the function of statistical analysis in cancer biology has grown increasingly significant in determining the various treatment alternatives. The length of time that elapses between the start of a specific time period and the occurrence of a chosen event is the subject of remission times or survival time data analysis. The purpose is usually to assess how different therapies effect remission time or survival time, and using easily available information about each patient adds to the uncertainty of the statistical distribution.

The goal of the current study is to determine how long cancer patients remain in remission after receiving EHLD. States and organizations perform tens of thousands of clinical trials annually to describe diseases and evaluate alternative therapies. The outcomes have a direct impact on how individuals are treated, thus it is critical to accurately assess the information presented in order to save both time and money. The majority of states use exploratory tools based on specified individuals to estimate the expected life or survival of patients. Acceptance sampling plans under indeterminacy would be one quality control methodology to save money and time when testing the patient's remission time or survival time. The oncologists are brainstorming to estimate the average remission time of the patients after attacked cancer due to their new method of treatment. In these situations the oncologists are paying attention to testing the null hypothesis of the average remission time of the patients is equal to the specified average remission time of the patients against the alternative hypothesis that the average remission time of the patients varies significantly. The null hypothesis could be rejected if the average remission time of the patients due to melanoma cancer, called as acceptance number of patients is greater than or equal to the specified average remission time of the patients due to melanoma cancer.

Numerous authors concentrate on studied single sampling plan (SSP) based time truncated life test for a variety of distributions. Some related article can also be explored^2–8^. More particulars related to SSP could available in^9^. The scheme of repetitive group acceptance sampling plan (RGASP) was first initiated by Sherman^10^. The improvement of single acceptance sampling plan is known as repetitive sampling plan for more details please explore^11–14^.

Although the aforementioned authors employed traditional statistics to examine the SSP and RASP, many real-world applications related to cancer patients' longevity may not. Recently, neutrosophic statistics have drawn the attention of more scholars in these circumstances. The development of more details about the neutrosophic logics, their quantification of determinacy, and their indeterminacy^15^. Various researchers considered neutrosophic logic for different real troubles and showed its competence as compared with fuzzy logic, for more information see^16–21^. The idea of neutrosophic statistics was given using the idea of neutrosophic logic^22–24^. Neutrosophic statistics commit information regarding the quantification of determinacy and measure of indeterminacy. Neutrosophic statistics become conventional statistics if no evidence is enrolled about the quantification of indeterminacy. The SSP using neutrosophic statistics is developed by Aslam^25,26^.

A fuzzy environment and the sample strategies at hand could not provide accounting data that is relevant to the measure of indeterminacy. Some works related to a single sampling plan using a fuzzy approach can also be explored^27–32^. More recently^33,34^ developed SSP and RASP to test average wind speed and COVID-19 patients for Weibull distribution under indeterminacy. When all of the observations in the sample or population are determined, the current sampling strategies based on classical statistics are employed. In practice, given the uncertainty environment, certain observations in the sample or population may be uncertain. In the latter scenario, the sampling plan for EHLD utilizing the RASP under classical statistics cannot be condemned. We were unable to identify a time-truncated sampling plan for EHLD under indeterminacy after searching the literature. We hope that the time-truncated sample strategy for EHLD under indeterminacy will be more useful for medical practitioners and industrial engineers when it comes to lot sizing in indeterminate contexts. As a result, we are motivated to examine RASP for EHLD in the presence of indeterminacy in order to calculate the average remission time. While testing to ascertain the average remission time, it is expected that the created sample design will have a lower ASN than the existing sampling designs.

In "Methodologies", we propose the RASP for EHLD under indeterminacy. A comparative study is given in "Comparative studies" and a real example based on the remission time of the patients due to melanoma cancer data is provided in "Applications of proposed plan for remission times of melanoma patients". In the end, concluding remarks, suggestions and future research works are demonstrated in "Conclusions".

## Methodologies

This section's goal is to provide an overview of the EHLD using neutrosophic statistics. This section will also show how to use the RASP to study the typical length of remission for melanoma cancer patients based on uncertain circumstances.

### Exponentiated half-logistic distribution under indeterminacy

We will provide a brief summary of the EHLD. The EHLD was acquainted and contemplated quite comprehensively by^35^, further^36^ studied for various acceptance sampling plans for this distribution. Suppose that $f\left({t}_{N}\right)=f\left({t}_{L}\right)+f\left({t}_{U}\right){I}_{N};{I}_{N}\epsilon \left[{I}_{L},{I}_{U}\right]$ be a neutrosophic probability density function (npdf) with determinate part $f\left({t}_{L}\right)$, indeterminate part $f\left({t}_{U}\right){I}_{N}$ and indeterminacy period ${I}_{N}\epsilon \left[{I}_{L},{I}_{U}\right]$ for more details refer^33^. Remember that ${t}_{N}\epsilon \left[{t}_{L},{t}_{U}\right]$ be a neutrosophic random variable (NRV) follows the npdf. The npdf is the oversimplification of pdf under conventional statistics. The anticipated neutrosophic form of $f\left({t}_{N}\right)\epsilon \left[f\left({t}_{L}\right),f\left({t}_{U}\right)\right]$ turns to pdf under classical statistics when ${I}_{L}$=0. Using this background, the npdf of the EHLD is outlined as under

1 $$f\left({t}_{N}\right)=\left\{\left(\frac{2\theta }{\sigma }\right)\frac{{\left(1-{e}^{-\frac{{t}_{N}}{\sigma }}\right)}^{\theta -1}}{{\left(1+{e}^{-\frac{{t}_{N}}{\sigma }}\right)}^{\theta +1}}\right\}+\left\{\left(\frac{2\theta }{\sigma }\right)\frac{{\left(1-{e}^{-\frac{{t}_{N}}{\sigma }}\right)}^{\theta -1}}{{\left(1+{e}^{-\frac{{t}_{N}}{\sigma }}\right)}^{\theta +1}}\right\}{I}_{N}; {I}_{N}\epsilon \left[{I}_{L},{I}_{U}\right],$$

where $\sigma$ and $\theta$ are scale and shape parameters, respectively. It is significant to note that the developed npdf of the EHLD is the oversimplification of pdf of the EHLD based on conventional statistics. The neutrosophic form of the npdf of the EHLD reduces to the EHLD when ${I}_{L}$=0. The neutrosophic cumulative distribution function (ncdf) of the EHLD is given by

2 $$F\left({x}_{N}\right)=\left\{{\left(\frac{1-{e}^{-\frac{{t}_{N}}{\sigma }}}{1+{e}^{-\frac{{t}_{N}}{\sigma }}}\right)}^{\theta }\right\}+\left\{{\left(\frac{1-{e}^{-\frac{{t}_{N}}{\sigma }}}{1+{e}^{-\frac{{t}_{N}}{\sigma }}}\right)}^{\theta }\right\}{I}_{N}; {I}_{N}\epsilon \left[{I}_{L},{I}_{U}\right].$$

The average lifetime of the NEHLD is given by

3 $${\mu }_{N}=\sigma \left[ln\left(\frac{1+{2}^{-1/\theta }}{1-{2}^{-1/\theta }}\right)\right]\left(1+{I}_{N}\right); {I}_{N}\epsilon \left[{I}_{L},{I}_{U}\right].$$

### Repetitive sampling plan under indeterminacy

The traditional RASP based on the truncated life test sampling scheme is initiated by^37^. The step-by-step procedure to adopt the repetitive acceptance sampling plan under indeterminacy is stated below:

Step 1: From a lot choose a sample of size *n*. Conduct a life testing for these sample for a pre-specified time say ${t}_{0}$. Indicate the average ${\mu }_{0N}$ and indeterminacy parameter ${I}_{N} \epsilon \left[{I}_{L},{I}_{U}\right]$.

Step 2: Accept ${H}_{0}:{\mu }_{N}={\mu }_{0N}$ if specified average quantity ${\mu }_{0N}$ is less than or equal to ${c}_{1}$ (i.e., ${\mu }_{0N}\le {c}_{1}$). If specified average quantity ${\mu }_{0N}$ is more than ${c}_{2}$ (i.e., ${\mu }_{0N}$ > ${c}_{2}$) then we reject ${H}_{0}:{\mu }_{N}={\mu }_{0N}$ and conclude the test, where ${c}_{1}\le {c}_{2}$.

Step 3: If ${{c}_{1}<\mu }_{0N}\le {c}_{2}$ then go to Step 1 and do again the entire procedure.

The developed RASP based on above indeterminacy methodology is consists of $n,{c}_{1},{c}_{2}$ and ${I}_{N}$, where ${I}_{N}\epsilon \left[{I}_{L},{I}_{U}\right]$ is known as uncertainty level and it is predetermined. RASP is a generalization of SSP under uncertainty studied in "Comparative studies". The proposed RASP is reduced to a SSP under uncertainty when ${c}_{1}={c}_{2}$. It is a convention to assume that ${t}_{0}=d{\mu }_{0}$ where $d$ is the termination factor. The operating characteristic (OC) function would be obtained based on lot acceptance probability for more details refer^10^ and it is defined as:

4 $$L\left( {p_{N} } \right) = \frac{{P_{a} \left( {p_{N} } \right)}}{{P_{a} \left( {p_{N} } \right) + P_{r} \left( {p_{N} } \right)}};\quad 0 < p_{N} < 1,$$

where $P_{a} \left( {p_{N} } \right)$ is the chance of accepting under ${H}_{0}:{\mu }_{N}={\mu }_{0N}$ whereas $P_{r} \left( {p_{N} } \right)$ is the chance of rejecting at ${H}_{0}:{\mu }_{N}={\mu }_{0N}$, these are obtained in the following expressions:

5 $$P_{a} \left( {p_{N} } \right) = \sum\limits_{i = 0}^{{c_{1} }} {\left( \begin{gathered} n \hfill \\ i \hfill \\ \end{gathered} \right)} p_{N}^{i} \left( {1 - p_{N} } \right)^{n - i} ,$$

6 $${\text{and}}\quad P_{r} \left( {p_{N} } \right) = 1 - \sum\limits_{i = 0}^{{c_{2} }} {\left( \begin{gathered} n \hfill \\ i \hfill \\ \end{gathered} \right)} p_{N}^{i} \left( {1 - p_{N} } \right)^{n - i} ,$$

where $p_{N}$ is the chance of rejecting ${H}_{0}:{\mu }_{N}={\mu }_{0N}$ and it is obtained from Eq. (2) and Eq. (3) and it is defined by

7 $${p}_{N}=\left\{{\left(\frac{1-\mathrm{exp}\left(-\frac{{{\rm d}\vartheta }\left(1+{I}_{N}\right)}{{\upmu }_{{\rm N}}/{\upmu }_{0{\rm N}}}\right)}{1+\mathrm{exp}\left(-\frac{\mathrm{d\vartheta }\left(1+{I}_{N}\right)}{{\upmu }_{{\rm N}}/{\upmu }_{0{\rm N}}}\right)}\right)}^{\theta }\right\}+\left\{{\left(\frac{1-\mathrm{exp}\left(-\frac{{{\rm d}\vartheta }\left(1+{I}_{N}\right)}{{\upmu }_{{\rm N}}/{\upmu }_{0{\rm N}}}\right)}{1+\mathrm{exp}\left(-\frac{{{\rm d}\vartheta }\left(1+{I}_{N}\right)}{{\upmu }_{{\rm N}}/{\upmu }_{0{\rm N}}}\right)}\right)}^{\theta }\right\}{I}_{N}.$$

Where $$\vartheta =ln\left(\frac{1+{2}^{-1/\theta }}{1-{2}^{-1/\theta }}\right)$$.

Using Eqs. (5) and (6) the Eq. (4) becomes

8 $$L\left( {p_{N} } \right) = \frac{{\sum\limits_{i = 0}^{{c_{1} }} {\left( \begin{gathered} n \hfill \\ i \hfill \\ \end{gathered} \right)} p_{N}^{i} \left( {1 - p_{N} } \right)^{n - i} }}{{\sum\limits_{i = 0}^{{c_{1} }} {\left( \begin{gathered} n \hfill \\ i \hfill \\ \end{gathered} \right)} p_{N}^{i} \left( {1 - p_{N} } \right)^{n - i} + 1 - \sum\limits_{i = 0}^{{c_{2} }} {\left( \begin{gathered} n \hfill \\ i \hfill \\ \end{gathered} \right)} p_{N}^{i} \left( {1 - p_{N} } \right)^{n - i} }};\quad 0 < p_{N} < 1.$$

The researcher is paying attention to concern the developed scheme to test ${H}_{0}:{\mu }_{N}={\mu }_{0N}$ such that the chance of accepting ${H}_{0}:{\mu }_{N}={\mu }_{0N}$ while it is true ought to be more than $1-\alpha$ ($\alpha$ is type-I ) for $\mu /{\mu }_{0}$ and the chance of accepting ${H}_{0}:{\mu }_{N}={\mu }_{0N}$ while it is wrong ought to be smaller than $\beta$ (type-II error) for ${\upmu }_{{\rm N}}/{\upmu }_{0{\rm N}}=1$. In producer opinion, the chance of approval should be greater than or equal to $1-\alpha$ at acceptable quality level (AQL), $p_{1N}$. In the same way, in consumer opinion the lot rejection chance ought to be less than or equal to $\beta$ at limiting quality level (LQL),$p_{2N}$. The intended quantities would be obtained by solving the following two inequalities simultaneously.

9 $$L\left( {p_{1N} \left| {{{\mu_{N} } \mathord{\left/ {\vphantom {{\mu_{N} } {\mu_{0N} }}} \right. \kern-0pt} {\mu_{0N} }}} \right.} \right) = \frac{{\sum\limits_{i = 0}^{{c_{1} }} {\left( \begin{gathered} n \hfill \\ i \hfill \\ \end{gathered} \right)} p_{1N}^{i} \left( {1 - p_{1N} } \right)^{n - i} }}{{\sum\limits_{i = 0}^{{c_{1} }} {\left( \begin{gathered} n \hfill \\ i \hfill \\ \end{gathered} \right)} p_{1N}^{i} \left( {1 - p_{1N} } \right)^{n - i} + 1 - \sum\limits_{i = 0}^{{c_{2} }} {\left( \begin{gathered} n \hfill \\ i \hfill \\ \end{gathered} \right)} p_{1N}^{i} \left( {1 - p_{1N} } \right)^{n - i} }} \ge 1 - \alpha ,$$

10 $$L\left( {p_{2N} \left| {{{\mu_{N} } \mathord{\left/ {\vphantom {{\mu_{N} } {\mu_{0N} = 1}}} \right. \kern-0pt} {\mu_{0N} = 1}}} \right.} \right) = \frac{{\sum\limits_{i = 0}^{{c_{1} }} {\left( \begin{gathered} n \hfill \\ i \hfill \\ \end{gathered} \right)} p_{2N}^{i} \left( {1 - p_{2N} } \right)^{n - i} }}{{\sum\limits_{i = 0}^{{c_{1} }} {\left( \begin{gathered} n \hfill \\ i \hfill \\ \end{gathered} \right)} p_{2N}^{i} \left( {1 - p_{2N} } \right)^{n - i} + 1 - \sum\limits_{i = 0}^{{c_{2} }} {\left( \begin{gathered} n \hfill \\ i \hfill \\ \end{gathered} \right)} p_{2N}^{i} \left( {1 - p_{2N} } \right)^{n - i} }} \le \beta .$$

where ${p}_{1N}$ and ${p}_{2N}$ are respectively given by

11 $${p}_{1N}=\left\{{\left(\frac{1-\mathrm{exp}\left(-{{\rm d}\vartheta }\left(1+{I}_{N}\right)\right)}{1+\mathrm{exp}\left(-{{\rm d}\vartheta }\left(1+{I}_{N}\right)\right)}\right)}^{\theta }\right\}+\left\{{\left(\frac{1-\mathrm{exp}\left(-{{\rm d}\vartheta }\left(1+{I}_{N}\right)\right)}{1+\mathrm{exp}\left(-{{\rm d}\vartheta }\left(1+{I}_{N}\right)\right)}\right)}^{\theta }\right\}{I}_{N},$$

and

12 $${p}_{2N}=\left\{{\left(\frac{1-\mathrm{exp}\left(-\frac{{{\rm d}\vartheta }\left(1+{I}_{N}\right)}{{\upmu }_{{\rm N}}/{\upmu }_{0{\rm N}}}\right)}{1+\mathrm{exp}\left(-\frac{{{\rm d}\vartheta }\left(1+{I}_{N}\right)}{{\upmu }_{{\rm N}}/{\upmu }_{0{\rm N}}}\right)}\right)}^{\theta }\right\}+\left\{{\left(\frac{1-\mathrm{exp}\left(-\frac{{{\rm d}\vartheta }\left(1+{I}_{N}\right)}{{\upmu }_{{\rm N}}/{\upmu }_{0{\rm N}}}\right)}{1+\mathrm{exp}\left(-\frac{{{\rm d}\vartheta }\left(1+{I}_{N}\right)}{{\upmu }_{{\rm N}}/{\upmu }_{0{\rm N}}}\right)}\right)}^{\theta }\right\}{I}_{N}.$$

The estimated intended quantities of the developed scheme should be minimizing the average sample number (ASN) at AQL. The ASN of the developed sampling scheme in terms of fraction defective ($p_{N}$) is given below:

13 $$ASN = \frac{n}{{P_{a} \left( {p_{N} } \right) + P_{r} \left( {p_{N} } \right)}} .$$

The intended quantities for the created method would therefore be determined by resolving the nonlinear programming problem for optimization shown below.

14 $$\begin{gathered} {\text{Minimize }}ASN(p_{1N} ) \hfill \\ {\text{subject to }} \hfill \\ L\left( {p_{1N} } \right) \ge 1 - \alpha \hfill \\ L\left( {p_{2N} } \right) \le \beta \hfill \\ 0 \le c_{1} \le c_{2} \hfill \\ {\text{where }}n,c_{1} ,c_{2} \in z. \hfill \\ \end{gathered}$$

The values of the intended quantities $\left\{n,{c}_{1}, {c}_{2}\right\}$ for various values of $\beta$ = {0.25, 0.10, 0.05}; $\alpha =0.10$; $d=\left\{0.5, 1.0\right\}$, ${\mu }_{N}/{\mu }_{0N}$={1.2, 1.3, 1.4, 1.5, 1.8, 2.0} and ${I}_{N}$= {0.0, 0.02, 0.04, 0.05} for shape parameter $\theta =\left\{{1,5}, 2.0, 1.0\right\}$ are presented in Tables 1, 2, 3, 4, 5 and 6. Tables 1 and 2 are shown for the EHLD for $\theta =1.5$, Tables 3 and 4 for $\theta =2.0$, Tables 5 and 6 for $\theta =1$ (half-logistic distribution). From these tables, we pointed out the below few points.

- When the values of $d$ increases from 0.5 to 1.0 the value of $ASN$ decreases.
- It is pointed out that if the shape parameter increases from $\theta =1 to \theta =2$ the values of $ASN$ decreases when other parameters are fixed.
- Further, it is observed that the indeterminacy value ${I}_{N}$ also showing a considerable effect to derogating the $ASN$.

**Table 1:** The RASP parameter of EHLD when $\alpha =0.10;\theta =1.5$ and $d=0.50$.

$\beta$	$\frac{{\upmu }_{{\rm N}}}{{\upmu }_{0{\rm N}}}$	${I}_{U}$ = 0.00					${I}_{U}$ = 0.02					${I}_{U}$ = 0.04					${I}_{U}$ = 0.05				
		$n$	${c}_{1}$	${c}_{2}$	$L\left({p}_{1}\right)$	ASN	$n$	${c}_{1}$	${c}_{2}$	$L\left({p}_{1}\right)$	ASN	$n$	${c}_{1}$	${c}_{2}$	$L\left({p}_{1}\right)$	ASN	$n$	${c}_{1}$	${c}_{2}$	$L\left({p}_{1}\right)$	ASN
0.25	1.2	106	17	23	0.9004	208.83	96	16	22	0.9005	195.67	101	18	24	0.9078	197.17	120	23	28	0.9014	190.20
	1.3	75	12	15	0.9040	107.11	77	13	16	0.9056	105.76	56	9	13	0.9115	100.56	67	12	15	0.9026	95.82
	1.4	25	2	6	0.9001	72.99	37	5	8	0.9075	63.99	28	3	7	0.9095	60.48	47	8	10	0.9042	56.91
	1.5	46	7	8	0.9009	52.61	36	5	7	0.9065	49.97	21	2	5	0.9069	45.62	33	5	7	0.9159	43.51
	1.8	16	1	3	0.9151	28.31	25	3	4	0.9015	27.72	14	1	3	0.9278	25.74	14	1	3	0.9211	24.28
	2.0	21	2	3	0.9092	25.46	19	2	3	0.9239	23.51	14	1	3	0.9597	22.74	9	0	2	0.9182	19.98
0.10	1.2	185	30	38	0.9044	312.70	177	30	38	0.9004	296.74	164	29	37	0.9004	280.51	124	21	30	0.9021	279.10
	1.3	93	13	19	0.9142	166.51	103	16	21	0.9011	152.34	93	15	20	0.9002	141.39	101	17	22	0.9070	140.13
	1.4	69	9	13	0.9003	102.15	51	6	11	0.9110	100.98	76	12	15	0.9015	96.30	61	9	13	0.9068	91.57
	1.5	56	7	10	0.9100	76.66	39	4	8	0.9037	70.54	37	4	8	0.9083	67.83	36	4	8	0.9114	66.64
	1.8	21	1	4	0.9169	45.14	28	2	5	0.9126	43.43	19	1	4	0.9199	37.70	19	1	4	0.9105	36.43
	2.0	15	0	3	0.9198	35.51	15	0	3	0.9017	32.92	24	2	4	0.9277	32.29	17	1	3	0.9055	25.65
0.05	1.2	227	36	46	0.9003	369.35	245	42	51	0.9011	354.85	201	35	45	0.9070	338.25	187	33	43	0.9025	323.41
	1.3	116	16	23	0.9063	189.82	116	17	24	0.9112	185.57	106	16	23	0.9032	172.04	93	14	21	0.9009	162.49
	1.4	71	8	14	0.9059	124.68	68	8	14	0.9023	118.23	64	8	14	0.9158	115.77	63	8	14	0.9098	111.86
	1.5	68	8	12	0.9105	92.26	59	7	11	0.9008	82.73	47	5	10	0.9148	80.10	40	4	9	0.9111	78.68
	1.8	38	3	6	0.9079	50.93	36	3	6	0.9113	48.59	35	3	6	0.9019	46.23	33	3	6	0.9199	45.46
	2.0	24	1	4	0.9228	38.80	24	1	4	0.9032	36.43	33	3	5	0.9181	35.07	32	3	5	0.9211	34.08

**Table 2:** The RASP parameter of EHLD when $\alpha =0.10;\theta =1.5$ and $d=1.00$.

$\beta$	$\frac{{\upmu }_{{\rm N}}}{{\upmu }_{0{\rm N}}}$	${I}_{U}$ = 0.00					${I}_{U}$ = 0.02					${I}_{U}$ = 0.04					${I}_{U}$ = 0.05				
		$n$	${c}_{1}$	${c}_{2}$	$L\left({p}_{1}\right)$	ASN	$n$	${c}_{1}$	${c}_{2}$	$L\left({p}_{1}\right)$	ASN	$n$	${c}_{1}$	${c}_{2}$	$L\left({p}_{1}\right)$	ASN	$n$	${c}_{1}$	${c}_{2}$	$L\left({p}_{1}\right)$	ASN
0.25	1.2	61	26	30	0.9033	93.44	41	17	22	0.9017	86.30	52	24	28	0.9099	84.02	51	24	28	0.9105	82.77
	1.3	41	17	19	0.9038	51.45	37	16	18	0.9061	49.34	33	14	17	0.9045	48.98	28	12	15	0.9081	44.59
	1.4	35	15	15	0.9038	35.00	26	11	12	0.9035	29.87	20	8	10	0.9025	28.19	24	10	12	0.9078	27.85
	1.5	15	5	7	0.9211	23.05	20	8	9	0.9152	22.39	18	7	9	0.9409	21.96	22	10	10	0.9007	21.00
	1.8	10	3	4	0.9177	12.58	6	1	3	0.9282	12.04	9	3	4	0.9267	12.53	11	4	5	0.9426	11.60
	2.0	8	2	3	0.9200	10.24	5	1	2	0.9017	9.13	7	2	3	0.9353	9.08	7	2	3	0.9298	8.17
0.10	1.2	75	30	37	0.9048	135.70	76	32	39	0.9142	131.72	76	34	40	0.9069	123.43	85	39	45	0.9025	121.24
	1.3	60	24	27	0.9015	73.35	50	20	24	0.9030	68.76	34	13	18	0.9066	63.35	27	10	15	0.9007	58.99
	1.4	25	8	12	0.9184	45.14	32	12	15	0.9154	43.81	31	12	15	0.9077	41.78	23	8	12	0.9028	39.89
	1.5	20	6	9	0.9071	32.97	26	9	12	0.9358	31.37	21	7	10	0.9041	30.17	20	7	10	0.9266	29.50
	1.8	14	3	6	0.9420	22.10	16	5	6	0.9012	18.82	16	4	7	0.9351	17.62	15	5	6	0.9026	16.71
	2.0	11	2	4	0.9035	14.51	10	2	4	0.9260	13.99	13	3	5	0.9176	12.69	13	4	5	0.9281	12.55
0.05	1.2	104	42	50	0.9059	160.85	75	30	39	0.9064	154.96	103	46	53	0.9026	145.06	86	38	46	0.9008	140.01
	1.3	55	20	26	0.9207	86.59	44	16	22	0.9050	76.29	40	15	21	0.9066	72.86	51	21	26	0.9018	71.50
	1.4	41	14	18	0.9015	53.70	28	9	14	0.9245	51.83	33	12	16	0.9005	46.06	30	11	15	0.9010	43.69
	1.5	27	8	12	0.9211	39.97	21	6	10	0.9090	36.88	20	6	10	0.9165	34.02	30	11	14	0.9193	32.08
	1.8	14	3	6	0.9420	22.10	11	2	5	0.9209	21.97	13	3	6	0.9387	20.17	13	3	6	0.9292	19.40
	2.0	12	1	5	0.9069	19.48	6	0	3	0.9014	17.17	10	2	4	0.9085	14.30	12	3	5	0.9415	13.40

**Table 3:** The RASP parameter of EHLD when $\alpha =0.10;\theta =2.0$ and $d=0.50$.

$\beta$	$\frac{{\upmu }_{{\rm N}}}{{\upmu }_{0{\rm N}}}$	${I}_{U }$= 0.00					${I}_{U}$ = 0.02					${I}_{U}$ = 0.04					${I}_{U}$= 0.05				
		$n$	${c}_{1}$	${c}_{2}$	$L\left({p}_{1}\right)$	ASN	$n$	${c}_{1}$	${c}_{2}$	$L\left({p}_{1}\right)$	ASN	$n$	${c}_{1}$	${c}_{2}$	$L\left({p}_{1}\right)$	ASN	$n$	${c}_{1}$	${c}_{2}$	$L\left({p}_{1}\right)$	ASN
0.25	1.2	101	13	17	0.9007	161.31	102	14	18	0.9029	159.47	76	10	15	0.9057	153.56	68	9	14	0.9055	144.91
	1.3	55	6	9	0.9080	88.66	52	6	9	0.9087	84.11	49	6	9	0.9121	79.92	42	5	8	0.9007	71.68
	1.4	40	4	6	0.9037	56.93	28	2	5	0.9017	56.79	26	2	5	0.9116	54.25	35	4	6	0.9026	49.93
	1.5	38	4	5	0.9040	44.69	36	4	5	0.9033	42.34	24	2	4	0.9090	37.98	29	3	5	0.9351	33.43
	1.8	17	1	2	0.9128	22.30	16	1	2	0.9139	21.05	16	1	2	0.9000	20.64	15	1	2	0.9097	19.64
	2.0	22	2	2	0.9178	22.00	15	0	2	0.9283	20.94	15	1	2	0.9515	19.82	15	1	2	0.9477	18.64
0.10	1.2	168	21	27	0.9040	253.99	159	21	27	0.9040	240.73	123	16	23	0.9040	227.49	147	21	27	0.9019	222.05
	1.3	82	8	13	0.9043	137.08	81	10	14	0.9068	125.48	59	6	11	0.9086	116.47	80	10	14	0.9094	116.20
	1.4	63	6	9	0.9075	88.81	61	7	10	0.9234	85.16	57	6	9	0.9014	77.76	38	3	7	0.9019	73.13
	1.5	41	3	6	0.9245	64.87	40	3	6	0.9104	60.89	30	2	5	0.9029	51.76	26	1	5	0.9033	50.69
	1.8	24	1	3	0.9274	35.58	23	1	3	0.9236	33.73	17	0	3	0.9386	38.55	22	1	3	0.9121	31.25
	2.0	16	0	2	0.9264	27.41	16	0	2	0.9120	25.99	14	0	2	0.9314	24.58	14	0	2	0.9251	23.95
0.05	1.2	186	22	30	0.9008	295.29	176	22	30	0.9010	279.93	173	23	31	0.9036	270.18	128	16	25	0.9059	266.49
	1.3	87	8	14	0.9081	154.48	83	8	14	0.9004	144.57	78	8	14	0.9080	138.96	83	9	15	0.9077	138.34
	1.4	70	6	10	0.9008	99.06	66	6	10	0.9030	93.94	44	3	8	0.9006	89.52	61	6	10	0.9013	86.60
	1.5	62	5	8	0.9051	78.28	46	3	7	0.9115	72.58	43	3	7	0.9187	69.41	43	3	7	0.9032	66.40
	1.8	21	0	3	0.9055	40.65	19	0	3	0.9215	39.35	19	0	3	0.9010	36.30	32	2	4	0.9169	31.22
	2.0	30	1	3	0.9204	37.18	28	1	3	0.9239	35.01	25	1	3	0.9386	32.65	24	1	3	0.9417	30.69

**Table 4:** The RASP parameter of EHLD when $\alpha =0.10;\theta =2.0$ and $d=1.00$.

$\beta$	$\frac{{\upmu }_{{\rm N}}}{{\upmu }_{0{\rm N}}}$	${I}_{U}$ = 0.00					${I}_{U}$ = 0.02					${I}_{U}$ = 0.04					${I}_{U}$ = 0.05				
		$n$	${c}_{1}$	${c}_{2}$	$L\left({p}_{1}\right)$	ASN	$n$	${c}_{1}$	${c}_{2}$	$L\left({p}_{1}\right)$	ASN	$n$	${c}_{1}$	${c}_{2}$	$L\left({p}_{1}\right)$	ASN	$n$	${c}_{1}$	${c}_{2}$	$L\left({p}_{1}\right)$	ASN
0.25	1.2	38	15	19	0.9018	67.42	34	14	18	0.9084	63.79	28	11	16	0.9023	62.11	32	14	18	0.9050	60.74
	1.3	26	10	12	0.9005	35.09	15	5	8	0.9018	31.15	26	11	13	0.9020	30.51	21	9	11	0.9049	29.78
	1.4	15	5	7	0.9185	25.05	9	2	5	0.9101	24.33	16	6	8	0.9216	23.52	16	6	8	0.9076	22.91
	1.5	11	3	5	0.9175	17.94	14	5	6	0.9041	16.77	13	5	6	0.9190	15.87	10	3	5	0.9090	14.21
	1.8	8	2	3	0.9267	13.24	5	1	2	0.9067	12.11	7	2	3	0.9379	11.24	9	3	4	0.9575	10.35
	2.0	6	1	2	0.9153	7.84	5	1	2	0.9457	7.11	9	3	3	0.9256	8.00	5	1	2	0.9312	6.85
0.1	1.2	72	29	34	0.9058	102.79	60	25	30	0.9032	91.84	42	17	23	0.9032	88.06	45	19	25	0.9126	86.16
	1.3	29	9	14	0.9007	54.64	26	9	13	0.9026	47.71	27	10	14	0.9091	45.84	36	15	18	0.9064	43.10
	1.4	20	6	9	0.9035	35.97	21	7	10	0.9272	32.45	18	6	9	0.9151	30.99	10	2	6	0.9053	29.67
	1.5	16	4	7	0.9125	25.13	15	4	7	0.9237	24.51	12	3	6	0.9120	21.72	17	6	8	0.9288	20.42
	1.8	11	2	4	0.9136	14.51	10	2	4	0.9318	13.93	7	1	3	0.9041	11.00	10	2	4	0.9022	10.85
	2.0	10	2	3	0.9089	12.33	7	1	3	0.9637	11.70	7	1	3	0.9547	11.00	9	2	3	0.9026	10.11
0.05	1.2	73	28	35	0.9084	117.25	70	28	35	0.9025	111.23	76	33	39	0.9074	107.19	53	22	29	0.9018	99.65
	1.3	34	11	16	0.9030	59.98	37	13	18	0.9139	58.15	43	17	21	0.9121	56.52	37	14	19	0.9105	55.42
	1.4	22	6	10	0.9001	36.05	21	6	10	0.9002	34.56	20	6	10	0.9039	33.38	33	13	15	0.9038	32.16
	1.5	18	4	8	0.9134	29.60	17	4	8	0.9209	28.78	26	9	11	0.9096	26.30	11	2	6	0.9034	25.63
	1.8	11	2	4	0.9136	15.51	15	4	5	0.9014	14.16	10	2	4	0.9131	13.19	10	2	4	0.9022	12.85
	2.0	13	2	4	0.9144	14.81	11	2	4	0.9520	13.79	10	2	4	0.9627	13.19	8	1	3	0.9068	10.40

**Table 5:** The RASP parameter of EHLD when $\alpha =0.10;\theta =1.0$ and $d=0.50$.

$\beta$	$\frac{{\upmu }_{{\rm N}}}{{\upmu }_{0{\rm N}}}$	${I}_{U}$= 0.00					${I}_{U }$= 0.02					${I}_{U}$ = 0.04					${I}_{U}$ = 0.05				
		$n$	${c}_{1}$	${c}_{2}$	$L\left({p}_{1}\right)$	ASN	$n$	${c}_{1}$	${c}_{2}$	$L\left({p}_{1}\right)$	ASN	$n$	${c}_{1}$	${c}_{2}$	$L\left({p}_{1}\right)$	ASN	$n$	${c}_{1}$	${c}_{2}$	$L\left({p}_{1}\right)$	ASN
0.25	1.2	186	43	50	0.9030	311.03	196	48	54	0.9014	293.86	165	41	48	0.9022	282.29	178	46	52	0.9006	271.19
	1.3	116	26	30	0.9031	163.20	82	18	23	0.9004	143.83	79	18	23	0.9008	138.92	81	19	24	0.9076	135.79
	1.4	79	17	20	0.9098	107.33	58	12	16	0.9088	97.91	52	11	15	0.9052	90.56	51	11	15	0.9064	89.08
	1.5	32	5	9	0.9011	70.76	45	9	12	0.9076	69.15	42	8	12	0.9127	66.76	25	4	8	0.9020	62.20
	1.8	23	3	6	0.9166	44.00	24	4	6	0.9047	35.32	23	4	6	0.9077	34.03	16	2	5	0.9276	33.19
	2.0	35	7	7	0.9047	35.80	20	3	5	0.9275	34.83	23	4	6	0.9464	32.03	15	2	4	0.9064	24.90
0.10	1.2	295	67	77	0.9013	461.41	272	64	74	0.9003	434.79	294	73	82	0.9004	424.77	222	54	65	0.9003	408.64
	1.3	152	32	39	0.9039	234.94	128	27	35	0.9068	229.81	137	31	38	0.9028	214.28	138	32	39	0.9084	212.76
	1.4	94	18	24	0.9088	153.43	101	21	26	0.9029	143.62	75	15	21	0.9008	142.48	86	18	24	0.9035	138.22
	1.5	63	11	16	0.9086	106.27	61	11	16	0.9032	101.46	63	12	17	0.9068	100.99	57	11	16	0.9151	97.57
	1.8	27	3	7	0.9053	55.64	26	3	7	0.9054	53.58	25	3	7	0.9071	51.76	29	4	8	0.9165	50.79
	2.0	26	3	6	0.9043	47.74	37	6	8	0.9129	45.28	24	3	6	0.9073	43.74	29	4	7	0.9032	41.49
0.05	1.2	307	67	81	0.9001	546.45	325	75	88	0.9005	520.11	305	73	86	0.9027	498.03	292	71	84	0.9008	482.95
	1.3	157	31	41	0.9030	279.87	178	38	47	0.9033	269.28	159	35	44	0.9050	252.75	156	35	44	0.9061	248.39
	1.4	101	18	26	0.9011	178.07	97	18	26	0.9044	172.19	108	22	29	0.9010	163.74	83	16	24	0.9094	161.56
	1.5	81	14	20	0.9066	125.97	58	9	16	0.9019	122.82	71	13	19	0.9008	115.88	74	14	20	0.9031	113.50
	1.8	49	7	11	0.9011	67.78	29	3	8	0.9120	65.80	40	6	10	0.9074	64.70	32	4	9	0.9213	63.95
	2.0	30	3	7	0.9016	52.11	28	3	7	0.9206	51.56	40	6	9	0.9106	50.39	39	6	9	0.9148	49.49

**Table 6:** The RASP parameter of EHLD when $\alpha =0.10;\theta =1.0$ and $d=1.00$.

$\beta$	$\frac{{\upmu }_{{\rm N}}}{{\upmu }_{0{\rm N}}}$	${I}_{U}$ = 0.00					${I}_{U}$ = 0.02					${I}_{U }$= 0.04					${I}_{U}$ = 0.05				
		$n$	${c}_{1}$	${c}_{2}$	$L\left({p}_{1}\right)$	ASN	$n$	${c}_{1}$	${c}_{2}$	$L\left({p}_{1}\right)$	ASN	$n$	${c}_{1}$	${c}_{2}$	$L\left({p}_{1}\right)$	ASN	$n$	${c}_{1}$	${c}_{2}$	$L\left({p}_{1}\right)$	ASN
0.25	1.2	83	36	42	0.9042	151.09	74	33	39	0.9011	140.93	86	41	46	0.9053	136.69	70	33	39	0.9106	135.15
	1.3	60	26	29	0.9119	81.70	54	24	27	0.9015	74.52	37	16	20	0.9009	66.95	49	23	26	0.9085	65.35
	1.4	23	8	12	0.9113	51.82	20	7	11	0.9118	49.15	31	13	16	0.9013	47.60	33	15	17	0.9043	43.23
	1.5	20	7	10	0.9174	38.80	27	11	13	0.9110	36.26	19	7	10	0.9040	35.24	28	12	14	0.9083	34.61
	1.8	16	5	7	0.9066	22.75	18	7	8	0.9244	21.26	10	3	5	0.9043	20.99	9	2	5	0.9132	19.22
	2.0	6	1	3	0.9113	18.24	11	3	5	0.9182	17.16	11	3	5	0.9002	16.38	10	3	5	0.9384	15.63
0.1	1.2	125	53	62	0.9040	225.83	108	47	56	0.9022	210.95	108	49	58	0.9124	210.66	136	65	72	0.9011	197.76
	1.3	77	31	37	0.9075	119.37	59	24	30	0.9016	104.31	59	25	31	0.9061	102.88	54	23	29	0.9003	97.71
	1.4	39	14	19	0.9040	70.39	40	15	20	0.9029	69.11	32	12	17	0.9021	63.30	43	18	22	0.9026	62.84
	1.5	32	11	15	0.9032	54.19	41	16	19	0.9078	52.78	45	19	21	0.9017	51.78	27	10	14	0.9066	45.67
	1.8	27	9	11	0.9104	32.14	23	8	10	0.9204	28.75	22	8	10	0.9268	27.79	18	6	9	0.9453	26.91
	2.0	24	8	9	0.9097	26.03	10	2	5	0.9299	22.47	10	2	5	0.9117	21.85	12	3	6	0.9355	20.65
0.05	1.2	168	71	82	0.9038	271.76	165	73	83	0.9016	251.99	147	67	77	0.9009	245.66	184	88	96	0.9043	241.28
	1.3	95	38	45	0.9065	137.44	97	41	47	0.9015	129.11	80	34	41	0.9041	121.60	61	25	33	0.9053	120.54
	1.4	57	21	27	0.9200	89.67	44	16	22	0.9015	81.76	59	24	29	0.9012	79.04	60	25	30	0.9075	78.65
	1.5	46	16	21	0.9159	65.93	40	14	19	0.9012	59.15	41	15	20	0.9016	58.25	38	14	19	0.9009	55.77
	1.8	17	4	8	0.9156	37.41	14	3	7	0.9011	35.74	22	7	10	0.9091	31.50	24	8	11	0.9165	30.94
	2.0	23	6	9	0.9020	28.22	20	6	8	0.9005	25.83	15	4	7	0.9313	23.19	19	6	8	0.9001	22.52

## Comparative studies

**Figure 1:**
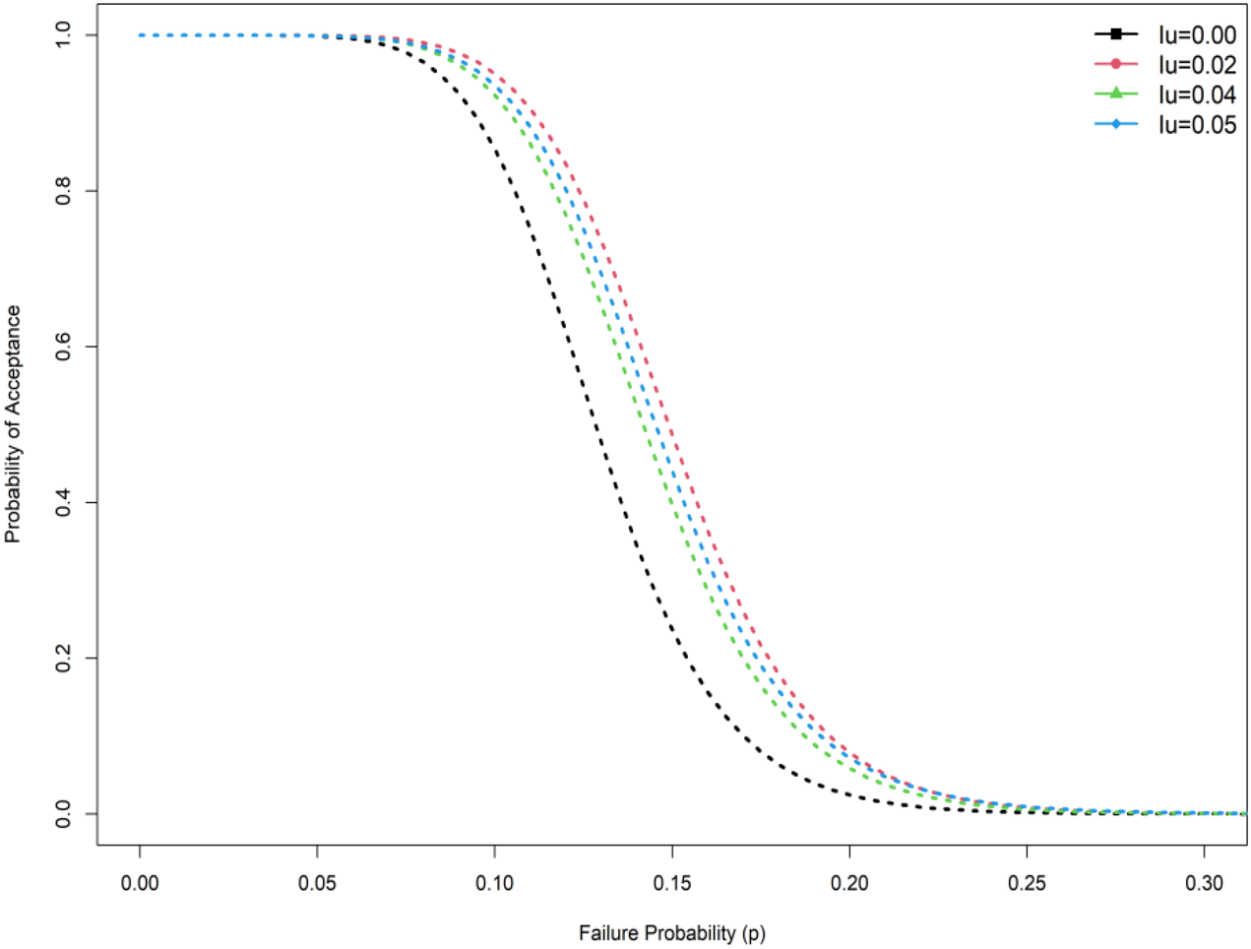
OC curve plan at different indeterminacy values.

**Figure 2:**
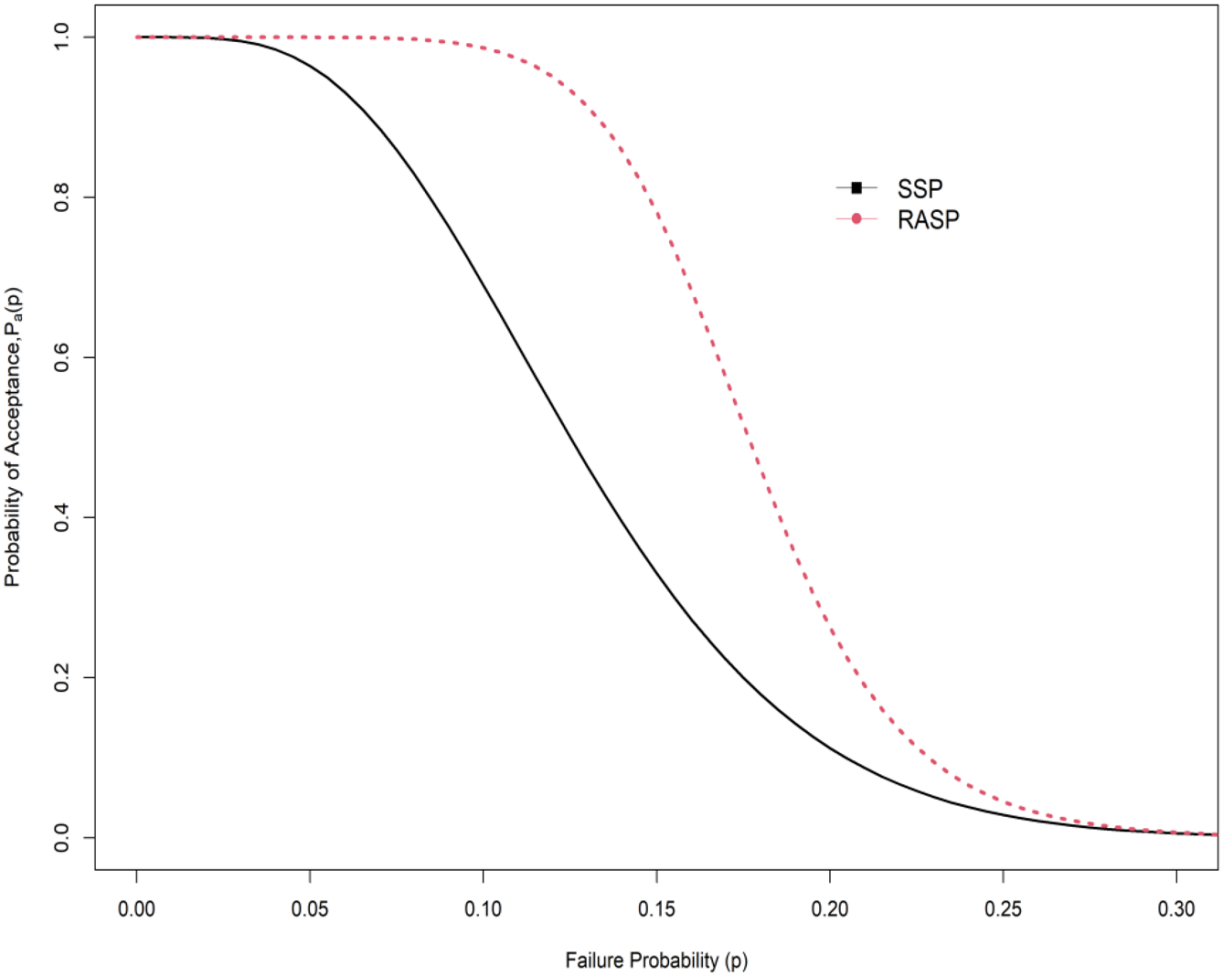
OC curves comparison between SSP and RASP under indeterminacy.

This section's goal is to examine the projected RASP's effectiveness in relation to ASN. The average hypothesis may be examined more affordably the lower the ASN. If no uncertainty or indeterminacy is established while remembering the average value, note that the sampling plan developed is an oversimplification of the plan based on conventional statistics. When ${I}_{N}$=0, the developed RSP becomes the on-hand sampling plan. In Tables 1, 2, 3, 4, 5 and 6 the first spell of column i.e. at ${I}_{N }$= 0 is the plan parameter of the traditional or existing RASP. From the results from the tables, we would conclude that the ASN is large in traditional RASP as compared with the proposed RASP. For example, when $\alpha =0.10, \beta =0.25,$ ${\upmu }_{{\rm N}}/{\upmu }_{0{\rm N}}$=1.3, $\theta$=1.5 and $d$=0.5 from Table 1, it can be seen that $ASN$=107.11 from the plan under classical statistics and $ASN$=95.82 for the projected RASP when ${I}_{N}$ = 0.05. Furthermore, when $\theta$=1 the EHLD becomes a half-logistic distribution (HLD), we have constructed Tables 5 and 6 for half-logistic distribution for comparison purpose. Table 5 depicts that EHLD shows less ASN as compared with HLD. For example when $\alpha =0.10,\beta =0.10,$ ${\upmu }_{{\rm N}}/{\upmu }_{0{\rm N}}$= 1.5, $d$= 0.5 and ${I}_{N}$= 0.04 the Table 5 shows that the ASN is 100.99 where as proposed plan values are *ASN* = 67.83 for $\theta$ = 1.5 and ASN = 51.76 for $\theta$ = 2.0. From this study, it is concluded that the projected plan under indeterminacy is efficient over the existing RASP under traditional statistics with respect to sample size. We have also compared our proposed RASP under indeterminacy with SSP under indeterminacy developed by^38^. The results show that RASP is superior to the SSP for same specific parameters. For example when $\alpha =0.10,\beta =0.10,$ ${\upmu }_{{\rm N}}/{\upmu }_{0{\rm N}}$ = 1.4, $d$= 0.5, ${I}_{N }$= 0.04 and $\theta$ = 1.5 the ASN in SSP is 105 whereas in RASP the ASN is 67.83. Operating characteristic (OC) curve of plan of the EHLD when $\alpha =0.10, \beta =0.10,\theta =2$, ${\upmu }_{{\rm N}}/{\upmu }_{0{\rm N}}$= 1.3 and $d$ = 0.5 is depicted in Fig. 1. From Fig. 1, we conclude that indeterminacy parameter shows significant effect on reduce the ASN. Therefore, the application of the proposed plan for testing the null hypothesis ${H}_{0}:{\mu }_{N}={\mu }_{0N}$ demands a lesser ASN as compared to the on hand plan. Moreover, the OC curve comparison between SSP and RASP is also displayed in Fig. 2. The OC curve in Fig. 2 also shows that RASP is superior to the SSP for the same specific parameters. The researchers advised as proposed RASP under uncertainty is more economical to apply in a medical study specifically for remission time of the patients due to melanoma cancer.

## Applications of proposed plan for remission times of melanoma patients

The present section deals with the postulation of the developed sampling scheme for the EHLD under the indeterminacy obtained by means of a real paradigm. This data set is picked out from^39^ and it constitutes the remission times, in months for 30 melanoma cancer patients at stages 2 to 4. For ready reference, the data is given below.

Remission time (months): 33.7, 3.9, 10.5, 5.4, 19.5, 23.8, 7.9, 16.9, 16.6, 33.7, 17.1, 8.0, 26.9, 21.4, 18.1, 16.0, 6.9, 11.0, 24.8, 23.0, 8.3, 10.8, 12.2, 12.5, 24.4, 7.7, 14.8, 8.2, 8.2 and 7.8.

Melanoma is a very dangerous kind of skin cancer which develops in the cells (melanocytes) that develop melanin and it creates the color change in the skin.

**Figure 3:**
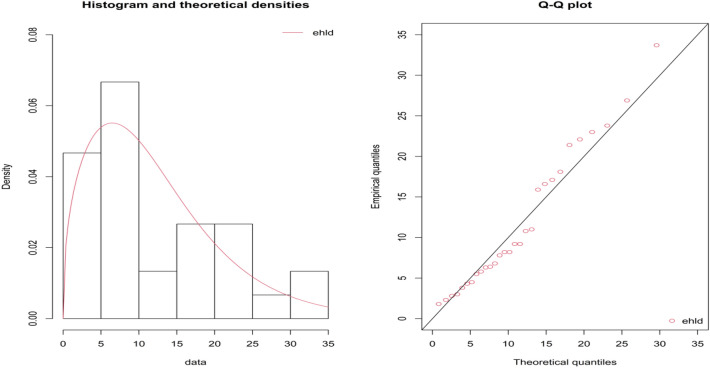
The empirical and theoretical pdf and Q-Q plots for the EHLD for the remission times of melanoma patients.

It is establish that the remission times of melanoma patients data comes from the EHLD with shape parameter $\widehat{\theta }=$ 1.4097 and scale parameter $\widehat{\sigma }=7.3811$ and the maximum distance between the real time data and the fitted of EHLD is found from the Kolmogorov–Smirnov test as 0.1324 and also the p-value is 0.6687. The demonstration of the goodness of fit for the given model is shown in Fig. 3, the empirical and theoretical cdfs and Q-Q plots for the EHLD for the remission times of melanoma patients’ data. In Tables 7 and 8 presented the plan quantities for the fitted shape parameter. It is assumed there is indeterminacy in measuring remission time and let it is 0.05. The measurements of remission time for cancer patients with respect to interval measures and fuzzy-type data sets were studied by various authors, for instance, refer^40–42^. For the proposed plan, the shape parameter is ${\widehat{\theta }}_{N}=(1+0.05)\times 1.4097 \approx 1.4802$ when ${I}_{U}$ = 0.05. Assume that a medical researcher would like to employ the developed RSP for EHLD under indeterminacy to guarantee the remission time of melanoma cancer patients is at least 6 months using the truncated life test for 3 months (thus *d* = 0.5). Suppose that medical researchers are paying attention to test ${H}_{0}:{\mu }_{N}=4.7893$ with the support of the developed RASP when ${I}_{U}$ = 0.05,$\alpha =0.10$, ${\upmu }_{{\rm N}}/{\upmu }_{0{\rm N}}$= 1.5, $d$ = 0.5 and $\beta$ = 0.10. From Table 7, it can be noted that* n* = 40, ${c}_{1}$= 5, ${c}_{2}$= 9 and ASN = 68.35. Thus, the RASP for EHLD under indeterminacy could be enforced in the following way: picking out a random sample of 40 melanoma cancer patients from the indoor group of patients, and conducting the truncated life test of remission time for 3 months. The developed RASP scheme could be adopted in the following way: hypothesis ${H}_{0}:{\mu }_{N}=4.7893$ will be accepted if the average remission time of melanoma cancer patients in 6 months is less than five patients, but a lot of patients should be rejected as soon as the remission time of melanoma cancer patients exceeds nine patients. Contrary, the experimentation could be repeated. From remission time data shows that seven patients before the average remission time of melanoma cancer patients of 4.7893. Therefore, the medical practitioners would have to repeat the entire procedure until accept/reject the hypothesis. Accordingly, it is competent that the developed sampling will be taken into consideration to check the typical length of remission for melanoma cancer patients based on the real application.

**Table 7:** The RASP parameter of EHLD when $\alpha =0.10;\theta =1.4097$ and $d=0.50$.

$\beta$	$\frac{{\upmu }_{{\rm N}}}{{\upmu }_{0{\rm N}}}$	${I}_{U}$ = 0.00					${I}_{U}$ = 0.02					${I}_{U}$ = 0.04					${I}_{U}$ = 0.05				
		$n$	${c}_{1}$	${c}_{2}$	$L\left({p}_{1}\right)$	ASN	$n$	${c}_{1}$	${c}_{2}$	$L\left({p}_{1}\right)$	ASN	$n$	${c}_{1}$	${c}_{2}$	$L\left({p}_{1}\right)$	ASN	$n$	${c}_{1}$	${c}_{2}$	$L\left({p}_{1}\right)$	ASN
0.25	1.2	116	20	26	0.9005	217.45	106	19	25	0.9000	204.05	106	20	26	0.9020	200.27	119	24	29	0.9000	189.62
	1.3	69	11	15	0.9089	116.38	61	10	14	0.9053	106.47	80	15	18	0.9022	105.26	57	10	14	0.9073	100.16
	1.4	29	3	7	0.9047	74.67	53	9	11	0.9020	69.85	43	7	10	0.9191	68.36	43	7	10	0.9054	66.76
	1.5	33	4	7	0.9168	58.61	26	3	6	0.9127	51.07	37	6	8	0.9178	50.89	24	3	6	0.9200	48.17
	1.8	24	3	4	0.9028	39.03	20	2	4	0.9272	33.75	27	4	5	0.9243	31.87	13	1	3	0.9195	24.27
	2.0	19	2	3	0.9113	28.57	15	1	3	0.9365	26.14	18	2	3	0.9001	22.04	13	1	3	0.9536	21.27
0.10	1.2	174	29	38	0.9029	330.56	225	42	49	0.9021	319.59	180	34	42	0.9020	295.56	176	34	42	0.9031	289.74
	1.3	111	17	23	0.9066	177.22	90	14	20	0.9045	158.70	86	14	20	0.9059	152.25	101	18	23	0.9031	148.08
	1.4	74	10	15	0.9131	118.86	73	11	15	0.9050	104.61	70	11	15	0.9030	99.88	56	8	13	0.9041	98.61
	1.5	56	7	11	0.9211	86.69	63	9	12	0.9006	80.69	54	8	11	0.9047	72.43	40	5	9	0.9003	68.35
	1.8	27	2	5	0.9121	44.42	19	1	4	0.9042	42.10	25	2	5	0.9059	41.36	23	2	5	0.9325	40.58
	2.0	35	4	5	0.9140	38.77	24	2	4	0.9123	36.49	20	1	4	0.9226	34.72	20	1	4	0.9138	33.62
0.05	1.2	239	40	51	0.9105	407.65	204	35	46	0.9006	371.88	226	42	52	0.9011	353.67	216	41	51	0.9040	345.06
	1.3	102	14	22	0.9027	200.61	103	15	23	0.9025	193.14	116	19	26	0.9028	180.35	86	13	21	0.9045	179.30
	1.4	73	9	15	0.9016	126.34	75	10	16	0.9103	125.13	61	8	14	0.9034	113.62	70	10	16	0.9130	112.57
	1.5	55	6	11	0.9089	92.87	58	7	12	0.9197	91.33	64	9	13	0.9094	86.40	49	6	11	0.9120	83.38
	1.8	35	3	6	0.9065	59.27	41	4	7	0.9052	52.29	22	1	5	0.9130	47.80	22	1	5	0.9009	45.69
	2.0	34	3	5	0.9061	40.96	22	1	4	0.9010	39.56	25	1	5	0.9187	36.49	20	1	4	0.9138	33.62

**Table 8:** The RASP parameter of EHLD when $\alpha =0.10;\theta =1.4097$ and $d=1.00$.

$\beta$	$\frac{{\upmu }_{{\rm N}}}{{\upmu }_{0{\rm N}}}$	${I}_{U}$ = 0.00					${I}_{U}$ = 0.02					${I}_{U}$ = 0.04					${I}_{U}$ = 0.05				
		$n$	${c}_{1}$	${c}_{2}$	$L\left({p}_{1}\right)$	ASN	$n$	${c}_{1}$	${c}_{2}$	$L\left({p}_{1}\right)$	ASN	$n$	${c}_{1}$	${c}_{2}$	$L\left({p}_{1}\right)$	ASN	$n$	${c}_{1}$	${c}_{2}$	$L\left({p}_{1}\right)$	ASN
0.25	1.2	79	35	38	0.9003	102.20	45	19	24	0.9005	90.81	56	26	30	0.9042	88.29	51	24	28	0.9025	83.08
	1.3	23	8	12	0.9080	51.82	20	7	11	0.9038	49.75	42	19	21	0.9000	48.98	32	14	17	0.9075	46.84
	1.4	34	14	15	0.9081	38.12	15	5	8	0.9159	36.36	22	9	11	0.9031	35.45	21	8	11	0.9064	34.66
	1.5	17	6	8	0.9296	25.52	17	6	8	0.9054	24.46	19	8	9	0.9122	23.41	18	7	9	0.9211	22.41
	1.8	10	3	4	0.9059	12.58	6	1	3	0.9177	12.03	9	3	4	0.9167	11.54	9	3	4	0.9088	11.41
	2.0	4	0	2	0.9126	10.67	10	2	4	0.9114	9.01	10	3	4	0.9219	8.02	6	1	3	0.9388	7.70
0.1	1.2	67	26	34	0.9047	149.95	94	41	47	0.9008	138.56	84	38	44	0.9043	129.66	84	38	45	0.9026	126.16
	1.3	48	18	23	0.9161	78.31	46	18	23	0.9196	75.87	57	25	28	0.9021	70.21	31	12	17	0.9027	62.80
	1.4	30	10	14	0.9060	48.26	28	9	14	0.9101	46.97	23	8	12	0.9094	43.39	25	9	13	0.9025	42.28
	1.5	31	11	13	0.9047	36.98	18	5	9	0.9124	35.32	34	14	15	0.9054	32.53	20	7	10	0.9148	30.62
	1.8	14	3	6	0.9286	22.10	14	3	6	0.9052	20.49	14	4	6	0.9149	18.23	14	4	6	0.9039	17.79
	2.0	13	3	5	0.9369	17.20	7	1	3	0.9024	16.77	9	2	4	0.9373	13.72	9	2	4	0.9304	13.06
0.05	1.2	98	39	48	0.9055	172.72	90	37	46	0.9001	162.68	76	32	41	0.9021	154.94	100	45	53	0.9002	152.01
	1.3	55	20	26	0.9044	86.59	55	21	27	0.9090	85.06	42	16	22	0.9007	75.46	57	24	29	0.9052	73.00
	1.4	42	14	19	0.9089	60.02	38	13	18	0.9043	55.76	27	9	14	0.9073	53.72	44	18	21	0.9045	52.37
	1.5	18	4	9	0.9031	42.58	26	8	12	0.9013	38.18	22	7	11	0.9214	36.65	22	7	11	0.9041	34.76
	1.8	14	3	6	0.9286	22.10	14	3	6	0.9052	20.79	13	3	6	0.9255	20.23	13	3	6	0.9144	19.47
	2.0	6	0	3	0.9044	18.70	12	2	5	0.9240	17.64	15	4	6	0.9387	17.10	15	4	6	0.9306	16.73

## Conclusions

In order to design an exponentiated half-logistic distribution based on indeterminacy for a time-truncated repetitive sampling strategy, a thorough investigation of melanoma cancer patients was conducted. The sample scheme parameters are determined for the identified values of the indeterminacy parameters. For simple reference, we have given lengthy tables including the values of the known indeterminacy constants. The developed sampling strategy is compared to the available conventional statistical strategies. The results show that the designed sampling plan is more cost-effective than the on-hand SSP under indeterminacy and conventional sampling plans. Furthermore, the proposed RASP under indeterminacy is more cost effective than the single sample strategy. It is also noticed that indeterminacy values play a vital role in ASN, when the indeterminacy quantities increase at that time the ASN quantity is decreased. Hence, the proposed sample strategies are convenient for researchers, particularly in medical experimentation, because medical experimentation requires more costly and qualified specialists. As a result, the created sampling strategy under indeterminacy is required to be valid for testing the average number of melanoma cancer patients. The real examples based on the melanoma cancer patients for developed sampling scheme under indeterminacy show a piece of evidence. The suggested sampling strategy for big data analytics could be applied to various scientific and technical disciplines. The next step in the research would be to develop multiple dependent state sampling plans and multiple dependent state repeating sampling plans for different lifetime distributions.

## Data Availability

Data is available in Supplementary Material file. Source of the data link is also provided.
